# *Impacto da Regulamentação do Ambiente Alimentar Escolar*: aspectos metodológicos e participação no primeiro ano de acompanhamento

**DOI:** 10.1590/0102-311XPT053925

**Published:** 2025-11-07

**Authors:** Laís Vargas Botelho, Larissa Loures Mendes, Letícia Ferreira Tavares, Paulo César Pereira de Castro, Juliana Souza Oliveira, Raquel Canuto, Raphaela Kistenmacker Pires, Letícia de Oliveira Cardoso

**Affiliations:** 1 Centro Multidisciplinar UFRJ-Macaé, Universidade Federal do Rio de Janeiro, Macaé, Brasil.; 2 Universidade Federal de Minas Gerais, Belo Horizonte, Brasil.; 3 Universidade Federal do Rio de Janeiro, Rio de Janeiro, Brasil.; 4 Universidade Federal de Pernambuco, Recife, Brasil.; 5 Universidade Federal do Rio Grande do Sul, Porto Alegre, Brasil.; 6 Universidade do Estado do Rio de Janeiro, Cabo Frio, Brasil.; 7 Escola Nacional de Saúde Pública Sergio Arouca, Fundação Oswaldo Cruz, Rio de Janeiro, Brasil.

**Keywords:** Acesso a Alimentos Saudáveis, Escolas, Percepção, Crianças, Adolescentes, Access to Healthy Foods, Schools, Perception, Children, Adolescents, Acceso a Alimentos Saludables, Escuelas, Percepción, Niños, Adolescentes

## Abstract

Este artigo descreve aspectos metodológicos e caracteriza a participação no primeiro ano do estudo *Impacto da Regulamentação do Ambiente Alimentar Escolar*. O estudo visa monitorar a comercialização de alimentos nas cantinas e no entorno de escolas privadas de três cidades brasileiras com distintos contextos regulatórios, bem como investigar a percepção dos escolares sobre a cantina. Trata-se de um estudo transversal repetido (2023/2024 e 2025), com amostras independentes, em Recife (Pernambuco), Niterói (Rio de Janeiro) e Porto Alegre (Rio Grande do Sul). Para o Módulo Cantina, planejou-se uma amostragem de escolas privadas de ensinos Fundamental e Médio, estratificada pelo porte escolar e com reposição inversa. Para o Módulo Ambulante, uma amostra de conveniência. E para o Módulo Aluno, uma amostra por conglomerados em três estágios. Os dois primeiros módulos replicam a metodologia do estudo *Comercialização de Alimentos em Escolas Brasileiras*. A primeira coleta de dados ocorreu entre setembro de 2023 e maio de 2024. Participaram 202 escolas e 532 alunos. Apenas três vendedores ambulantes foram localizados. As taxas de participação escolar foram de 47,8% em Porto Alegre, 53,9% em Niterói e 69,9% em Recife. Houve resistência das escolas em receber a pesquisa, o que prolongou a coleta de dados e exigiu adaptações metodológicas. O artigo discute os desafios enfrentados no trabalho de campo e os ajustes metodológicos para o segundo ano de acompanhamento, trazendo recomendações para futuros estudos no ambiente escolar. Este projeto é fruto da colaboração entre academia e sociedade civil organizada, que demandou informações para apoiar ações de *advocacy* pela alimentação saudável na escola. As evidências geradas poderão subsidiar a formulação e o aprimoramento de políticas de proteção do ambiente alimentar escolar em outras cidades e níveis governamentais.

## Abstract (en)

This article describes methodological aspects and characterizes participation in the first year of the study *Impact of Regulation of the School Food Environment*. The aim was to monitor food sales in and around private school canteen in three Brazilian cities with different regulatory contexts and investigate the perceptions of students with regards to canteen. A repeated cross-sectional study (2023/2024 and 2025) was conducted with independent samples in the cities of Recife (Pernambuco State), Niterói (Rio de Janeiro State), and Porto Alegre (Rio Grande do Sul State). For the Canteen Module, sampling involved private primary schools and high schools stratified by school size and with inverse replacement. A convenience sample was used for the Street Vendor Module and 3-stage cluster sampling was used for the Student Module. The first two modules replicate the method of the *Food Commercialization in Brazilian Schools* study. The first data collection process took place between September 2023 and May 2024. Two hundred two schools and 532 students participated. Only three street vendors were located. School participation rates were 47.8% in Porto Alegre, 53.9% in Niterói, and 69.9% in Recife. Schools were reluctant to participate in the survey, which prolonged the data collection process. This article discusses the challenges faced during fieldwork and the methodological adjustments required for the second year of monitoring, offering recommendations for future studies in the school setting. This project is the result of a collaboration between academia and organized civil society, which required information to support advocacy for healthy eating at schools. The evidence generated could assist in the formulation and improvement of protection policies for the school food environment in other cities and on other governmental levels.

## Abstract (es)

Este artículo describe aspectos metodológicos y caracteriza la participación en el primer año del estudio *Impacto de la Regulación del Entorno Alimentario Escolar*. El estudio monitoriza la venta de alimentos en los establecimientos que comercializan sus productos alimenticios dentro de estos centros educativos y en el entorno de escuelas privadas de tres ciudades brasileñas con distintos contextos regulatorios, así como investigar la percepción de la comunidad escolar sobre estos cantina. Es un estudio transversal repetido (2023/2024 y 2025), con muestras independientes, en Recife (Pernambuco), Niterói (Río de Janeiro) y Porto Alegre (Río Grande do Sul). Para el Módulo Cantina, se planificó un muestreo de escuelas privadas de enseñanza primaria y secundaria, estratificado por tamaño escolar y con reposición inversa; para el Módulo Vendedor Ambulante, una muestra de conveniencia; y para el Módulo Alumno, una muestra por conglomerados en tres etapas. Los dos primeros módulos replican la metodología del estudio *Comercialización de Alimentos en Escuelas Brasileñas*. La primera recopilación de datos ocurrió entre septiembre de 2023 y mayo de 2024. Participaron 202 escuelas y 532 alumnos. Se localizaron tres vendedores ambulantes. Las tasas de participación escolar fueron del 47,8% en Porto Alegre, del 53,9% en Niterói y del 69,9% en Recife. Las escuelas mostraron resistencia a aceptar la encuesta, lo que prolongó la recopilación de datos y exigió adaptaciones metodológicas. Se discuten los desafíos enfrentados en el trabajo de campo y los ajustes metodológicos para el segundo año de seguimiento, brindando recomendaciones para futuros estudios en el ámbito escolar. Este proyecto es fruto de la colaboración entre el mundo académico y la sociedad civil organizada, que solicitó información para respaldar las acciones de promoción de una alimentación saludable en las escuelas. Las pruebas generadas podrán servir de base para la formulación y mejora de políticas de protección del entorno alimentario escolar en otras ciudades y niveles gubernamentales.

## Introdução

O ambiente escolar pode promover ou não uma alimentação saudável, dependendo de suas características que afetam a acessibilidade, desejabilidade e conveniência dos alimentos saudáveis e não saudáveis ^1^. No Brasil, o ambiente alimentar de escolas públicas e privadas difere em seu potencial de promover práticas alimentares saudáveis, refletindo escolhas políticas distintas ^2^. Enquanto as escolas públicas oferecem refeições gratuitas e acessíveis por meio do Programa Nacional de Alimentação Escolar (PNAE) ^3^, nas privadas, as cantinas comerciais são a principal fonte alimentar ^4^.

Segundo a *Pesquisa Nacional de Saúde do Escolar* (PeNSE) de 2019, o percentual de escolares de 13 a 17 anos expostos a cantinas comerciais era 2,8 vezes maior nas escolas privadas do que nas públicas (88,3% *vs.* 31,4%) ^4^. Esse cenário preocupa, pois a comercialização de alimentos ultraprocessados, como refrigerantes, salgados fritos, biscoitos, salgadinhos embalados, sanduíches e pizzas, é frequente em cantinas escolares, especialmente na rede privada ^4^ ^,^ ^5^ ^,^ ^6^. Na ausência de uma lei federal sobre a comercialização de alimentos no ambiente escolar, estados e municípios apresentam contextos regulatórios variados, que vão da ausência de regulamentação a leis bem consolidadas. Assim, apenas localidades com regulamentação própria oferecem alguma proteção ao ambiente alimentar das escolas privadas ^7^.

A exposição a pontos alternativos de venda de alimentos nos arredores das escolas é um desafio tanto para a rede pública quanto para a privada. Embora mais frequente entre estudantes da rede pública (54,8%), o percentual de alunos de escolas privadas com acesso a essas opções também é elevado (39%) ^4^. Nesse contexto, acredita-se que estudantes da rede privada enfrentam maior exposição a ambientes alimentares obesogênicos, em comparação aos de escolas públicas, devido à maior dependência de cantinas comerciais e pontos de venda informal de alimentos ^2^ ^,^ ^6^.

A pesquisa *Comercialização de Alimentos em Escolas Brasileiras* (Caeb), apresentada anteriormente em CSP ^8^, é um estudo transversal de base escolar realizado entre 2022 e 2024, com o objetivo de avaliar a venda de alimentos e bebidas em cantinas comerciais e pontos de venda informais no entorno de escolas privadas de ensinos Fundamental e Médio. O estudo-piloto ocorreu em Niterói (Rio de Janeiro), seguido da coleta de dados em 2.241 cantinas escolares e 700 vendedores ambulantes nas 26 capitais brasileiras e no Distrito Federal ^8^.

O estudo *Impacto da Regulamentação do Ambiente Alimentar Escolar* foi concebido para repetir a pesquisa do Caeb nos dois anos subsequentes, monitorando a comercialização de alimentos e avaliando a relação entre possíveis mudanças e o contexto regulatório local em três das cidades anteriormente avaliadas: Recife (Pernambuco), Niterói e Porto Alegre (Rio Grande do Sul), que apresentam diferentes cenários regulatórios.

O estudo também adicionou um módulo de pesquisa com os alunos, que investiga a percepção sobre a cantina escolar nesses diferentes contextos regulatórios. Além disso, são objetivos secundários analisar o consumo alimentar e o estado nutricional autorreferido dos estudantes e sua associação com a saudabilidade da cantina.

Este artigo descreve a metodologia do estudo *Impacto da Regulamentação do Ambiente Alimentar Escolar* e caracteriza a participação no primeiro ano de acompanhamento, abordando desafios e adaptações no trabalho de campo. O relato visa apoiar análises futuras dos dados obtidos e orientar outros pesquisadores interessados no monitoramento da comercialização de alimentos em escolas.

## Aspectos metodológicos

### Desenho de estudo e coleta de dados

O estudo *Impacto da Regulamentação do Ambiente Alimentar Escolar* é um estudo transversal repetido, configurando um painel descontínuo, planejado para ocorrer no segundo semestre de 2023 e 2024 nas cidades de Recife, Niterói e Porto Alegre, com coletas de dados em amostras independentes.

O estudo compreende três módulos: (i) Módulo Cantina, uma auditoria sobre a comercialização de alimentos e bebidas nas cantinas escolares; (ii) Módulo Ambulante, que avalia pontos de venda informais no entorno das escolas; e (iii) Módulo Aluno, um *websurvey* aplicado a estudantes de uma subamostra de escolas participantes do Módulo Cantina.

### Contexto

As cidades de Recife, Niterói e Porto Alegre foram selecionadas para o acompanhamento devido aos seus diferentes contextos regulatórios, pois se encontram em diferentes estágios de implementação de medidas para regulamentar o ambiente alimentar escolar, com dispositivos legais existentes ou propostos de escopos variados (Material Suplementar - Quadro S1; https://cadernos.ensp.fiocruz.br/static//arquivo/suppl-e00053925_4814.pdf). A escolha também foi direcionada pela demanda de uma organização da sociedade civil de interesse público (OSCIP) parceira por informações para subsidiar ações de *advocacy* pela infância saudável junto aos governantes locais em Niterói e Recife.

O contexto regulatório de Recife era caracterizado pela ausência de leis municipais ou estaduais voltadas à promoção da alimentação saudável nas escolas. Havia apenas um Projeto de Lei e um Substitutivo, apresentados à Câmara Municipal do Recife em 2022 pela referida OSCIP ^9^.

Niterói estava sob leis municipais e estaduais que proibiam a comercialização e a publicidade de guloseimas, bebidas açucaradas e alimentos com teores de nutrientes acima de limites críticos (Material Suplementar - Quadro S1; https://cadernos.ensp.fiocruz.br/static//arquivo/suppl-e00053925_4814.pdf). No entanto, essas normas eram pouco conhecidas, não haviam sido regulamentadas por decreto e não contavam com fiscalização. Em janeiro de 2023, entre a coleta de dados do Caeb e a primeira coleta deste estudo, a *Lei Municipal nº 3.766* ^10^ reformulou a legislação anterior, tornando explícito o veto aos alimentos ultraprocessados em escolas públicas e privadas. No entanto, o decreto regulamentador dessa nova legislação foi publicado apenas após a conclusão da primeira etapa do presente estudo. Destaca-se que a mesma OSCIP atuante em Recife teve um papel central nessas mudanças, utilizando dados do Caeb e achados preliminares deste estudo para embasar ações de *advocacy*.

Porto Alegre, por sua vez, foi considerada estratégica para comparação por ter uma legislação mais consolidada, sendo reconhecida como referência nacional na promoção da alimentação adequada e saudável nas escolas. Segundo Rocha et al. ^7^, é uma das poucas capitais brasileiras protegidas por um conjunto de leis e decretos municipais e estaduais considerados plenamente capazes de cumprir esse papel. Embora esses dispositivos não mencionem explicitamente o termo “ultraprocessado”, fazem referência ao *Guia Alimentar para a População Brasileira* e definem os responsáveis pela fiscalização.

### População e amostragem

#### Módulo Cantina

O universo amostral do Módulo Cantina foi definido a partir do Catálogo de Escolas do Instituto Nacional de Estudos e Pesquisas Educacionais Anísio Teixeira (INEP), do Ministério da Educação. Como a plataforma do INEP não informa a data de atualização do catálogo, a listagem foi extraída próximo ao início do trabalho de campo para garantir maior atualidade.

Foram incluídas escolas privadas que atendiam aos seguintes critérios: (i) ofertavam Ensino Fundamental e/ou Médio; (ii) possuíam pelo menos 50 matrículas ativas; e (iii) contavam com cantina. Escolas menores ou exclusivas de Ensino Infantil foram excluídas, pois, conforme verificado no Caeb, a maioria não possuía cantina ou adotava outros modelos de lanches.

Como não há informações disponíveis sobre a presença de cantinas, o universo amostral inicial incluiu todas as escolas que atendiam aos dois primeiros critérios: 129 em Niterói, 102 em Porto Alegre e 331 em Recife. Em cada cidade, a seleção foi feita por amostragem aleatória simples, estratificada proporcionalmente à distribuição das escolas segundo o porte (51-200, 201-500, 501-1.000 ou > 1.000 alunos). A elegibilidade foi verificada preferencialmente por telefone ou e-mail e, quando necessário, site, mídias sociais ou visitas presenciais.

A amostra foi calculada estimando-se que 60% das escolas privadas de ensino fundamental e/ou médio possuíam cantina, com base na PeNSE 2015 ^11^ e na triagem telefônica do Caeb ^8^. Considerou-se uma prevalência de 50% a fim de maximizar a variabilidade da amostra e assegurar poder estatístico robusto para avaliar múltiplos aspectos, erro amostral de 5% e nível de 95% de significância, resultando em 67 escolas em Niterói, 54 em Porto Alegre e 138 em Recife. Utilizou-se a amostragem inversa para garantir a aleatoriedade na reposição de escolas inelegíveis, desativadas, recusantes ou sem contato ^12^. As listas de reposição foram esgotadas, de modo que todas as escolas foram triadas quanto à elegibilidade e convidadas a participar.

#### Módulo Ambulante

Como não existe registro formal confiável do comércio de rua, o universo amostral não poderia ser previamente conhecido, então foi planejada uma amostra não probabilística atrelada ao delineamento amostral das cantinas. Todos os ambulantes presentes no entorno imediato de cada escola participante no momento da visita foram considerados elegíveis. O entorno foi definido como o passeio ou calçada que circunda toda a escola, bem como a calçada do lado oposto à portaria de entrada ou saída.

#### Módulo Aluno

No Módulo Aluno, adotou-se uma amostragem por conglomerados em três estágios. Primeiro, foram selecionadas 15 escolas por cidade, seguindo a mesma estratificação proporcional ao porte adotada do Módulo Cantina. No segundo, sortearam-se três turmas do 7º ano do Ensino Fundamental ao 2º ano do Ensino Médio (12 a 17 anos). Por fim, todos os estudantes dessas turmas foram convidados a participar.

A estimativa do tamanho amostral baseou-se em um estudo que avaliou a relação entre a oferta de alimentos não saudáveis na cantina e seu consumo, com razões de chances entre 1,2 e 1,4 ^5^. Considerou-se uma diferença relativa de 30% em relação a uma prevalência de 50%, com nível de 95% de confiança, poder de 80% e efeito do plano amostral de 1,2. Assim, estimaram-se 450 alunos por cidade, totalizando 1.350. Para alcançar essa amostra, calculou-se a necessidade de convidar 4.500 estudantes, assumindo uma média de 30 alunos por turma e uma taxa de adesão conservadora de 30% ^13^.

### Composição da equipe, treinamento e coleta de dados

O estudo é coordenado pela Escola Nacional de Saúde Pública Sergio Arouca, Fundação Oswaldo Cruz (Ensp/FIOCRUZ), com equipe técnica composta por pesquisadores dessa instituição e das Universidades Federais do Rio de Janeiro e de Minas Gerais. Cada cidade conta com uma coordenadora, uma supervisora de campo e três entrevistadores (Material Suplementar - Figura S1; https://cadernos.ensp.fiocruz.br/static//arquivo/suppl-e00053925_4814.pdf).

A equipe técnica elaborou um protocolo ^14^ detalhando as etapas dos três módulos e as atribuições de cada função, além de uma planilha padronizada para controle da triagem e substituição de escolas.

O treinamento dos entrevistadores envolveu uma apresentação remota de 40 minutos e duas horas de simulação em pontos de venda reais. Foi disponibilizada uma versão revisada do manual de campo do Caeb ^15^ e um manual ^16^ de uso do software Research Electronic Data Capture (REDCap; https://project-redcap.org/), acompanhado por um vídeo instrucional de três minutos ^17^.

A coleta de dados dos Módulos Cantina e Ambulante se deu por observação direta e entrevistas com proprietários dos pontos de venda, utilizando o REDCap instalado nos *smartphones* dos entrevistadores.

No Módulo Aluno, a coleta de dados foi realizada digitalmente, utilizando um questionário autopreenchível no Google Forms (https://docs.google.com/forms). Como o público-alvo eram adolescentes, foi necessário obter o consentimento dos responsáveis e assentimento dos alunos. Para evitar múltiplas visitas às escolas e reduzir custos, optou-se pela coleta assíncrona por meio de um *websurvey*. Um *kit* digital foi desenvolvido e enviado pelas escolas aos responsáveis, contendo um PDF informativo sobre a pesquisa, links para os registros de consentimento e assentimento, e para o *Questionário do Aluno*. Também foram enviados links para postagens em redes sociais, reportagens sobre a pesquisa e o site do Caeb ^15^. Durante as visitas, as equipes usaram crachás e uniformes padronizados.

### Instrumentos e variáveis

#### Módulo Cantina

No Módulo Cantina, os dados foram coletados com o *Instrumento para Avaliação da Comercialização de Alimentos em Cantinas de Escolas* ^15^, desenvolvido pela equipe técnica do Caeb tendo a classificação NOVA ^18^ como referência. O instrumento foi avaliado quanto à validade de conteúdo, confiabilidade interobservador e teste-reteste, apresentando concordância satisfatória no estudo de validade e classificações entre boa e excelente nos testes de confiabilidade ^15^.

O instrumento inclui as seções: (i) identificação e caracterização; (ii) comercialização de alimentos (preço, variedade, tamanho, estratégia de venda); e (iii) publicidade ^8^. As variáveis coletadas estão descritas no Material Suplementar (Quadro S2; https://cadernos.ensp.fiocruz.br/static//arquivo/suppl-e00053925_4814.pdf. A lista de verificação de alimentos abrange 50 subgrupos, divididos em 21 alimentos *in natura*, minimamente processados, processados e preparações culinárias à base desses alimentos e 29 alimentos ultraprocessados e suas preparações (Material Suplementar - Quadro S3; https://cadernos.ensp.fiocruz.br/static//arquivo/suppl-e00053925_4814.pdf).

#### Módulo Ambulante

A coleta de dados no entorno escolar foi realizada com o *Instrumento para Avaliação do Comércio Informal de Alimentos no Entorno de Escolas* ^15^, uma versão do instrumento utilizado nas cantinas, adaptado às particularidades do comércio ambulante, incluindo dados administrativos (cadastro em órgãos públicos, horário de funcionamento), tipo de estrutura (carrinho, barraca, *food truck*, banca), localização e condições higiênico-sanitárias ^8^. A coleta ocorreu em dias úteis, dentro do intervalo entre 7h e 17h, sendo realizada com os vendedores presentes no local no momento da visita da equipe de pesquisa a cada escola, antes ou após a aplicação do Módulo Cantina.

#### Módulo Aluno

O *Questionário do Aluno* ^15^ foi desenvolvido pela equipe técnica do presente estudo. Composto por 87 questões, distribuídas em quatro blocos (Material Suplementar - Quadro S4; https://cadernos.ensp.fiocruz.br/static//arquivo/suppl-e00053925_4814.pdf). O instrumento aborda quatro blocos: (1) informações gerais; (2) percepção sobre a saudabilidade da cantina escolar; (3) alimentação; e (4) atividade física. As questões dos blocos 1, 3 e 4 foram baseadas ou adaptadas dos questionários da PeNSE 2019 ^4^, da *Pesquisa Nacional de Saúde* (PNS) de 2019 ^19^ e do *Sistema de Vigilância de Fatores de Risco e Proteção para Doenças Crônicas por Inquérito Telefônico* (Vigitel) de 2021 ^20^. O bloco 2, referente à percepção sobre a cantina escolar, foi desenvolvido especificamente para este estudo.

#### Desenvolvimento do *Questionário de Percepção sobre a Cantina Escolar*

Foi realizada uma revisão de escopo da literatura para identificar instrumentos completos ou questões individuais que abordassem a percepção dos estudantes sobre o ambiente alimentar escolar ^21^. Não foram encontrados instrumentos apropriados, mas foram recuperados estudos relevantes sobre as atitudes dos estudantes. A atitude é um construto da dimensão psicológica da percepção, definido como uma predisposição mental que influencia as ações, baseada no julgamento pessoal de algo como positivo ou negativo ^22^.

Por exemplo, Gosliner et al. ^23^ investigaram atitudes dos estudantes em relação a cenários potenciais com a pergunta: “*How important is it to you to be able to buy the following food items at school?*” [Quão importante é para você poder comprar os seguintes itens alimentares na escola?], respondida em escala Likert para avaliar a importância atribuída ao acesso a determinados alimentos na escola. Já Hermans et al. ^24^ avaliaram a percepção sobre a realidade concreta do ambiente alimentar escolar vivenciado por meio da pergunta: “*What score would you give the food and drinks assortment of your school canteen?*” [Que nota você daria à variedade de alimentos e bebidas do refeitório da sua escola?], que busca captar diretamente a experiência prática dos estudantes com a oferta de alimentos na escola.

Devido à inexistência de um instrumento apropriado e ao suporte limitado da literatura para sua construção, foi desenvolvido um questionário específico para o Módulo Aluno e decidiu-se enfocar a cantina, ao invés do construto ambiente alimentar escolar, como um todo. O questionário incluiu questões tanto sobre a percepção dos estudantes em relação a aspectos objetivos referentes às cantinas ^25^ quanto sobre suas atitudes frente a cenários potenciais ^23^. Os itens 4 a 11, formulados como “É importante que a cantina da minha escola venda [nome do alimento ou bebida]”, foram baseados na questão de Gosliner et al. ^23^.

A versão inicial do instrumento desenvolvido consistia em 28 itens: uma pergunta de triagem sobre a frequência de uso da cantina e 27 afirmações, para as quais os respondentes indicavam seu grau de concordância em uma escala Likert de cinco pontos, variando de “discordo totalmente” (1) a “concordo totalmente” (5).

Quatro membros da equipe técnica, com *expertise* em nutrição, epidemiologia, saúde coletiva e ambiente alimentar, utilizaram a mesma escala Likert de concordância para avaliar a versão do questionário quanto à relevância e clareza dos itens, além da adequação das opções de resposta. Também foi realizada uma avaliação qualitativa por meio de comentários abertos. Itens com avaliação mediana ou baixa (nota 3 ou inferior) ou que receberam sugestões dos especialistas foram revisados e, quando necessário, modificados, como a exclusão do item 4 e a simplificação de termos - por exemplo, a substituição de “grande variedade” por “várias opções” (Material Suplementar - Quadro S5; https://cadernos.ensp.fiocruz.br/static//arquivo/suppl-e00053925_4814.pdf).

O questionário revisado foi pré-testado em duas turmas de escolas niteroienses com características semelhantes à amostra do estudo. Os alunos preencheram o questionário impresso e forneceram observações detalhadas. Também foi realizado um grupo focal para identificar facilidades e dificuldades no preenchimento do instrumento. As principais modificações foram a alteração de “É importante” para “Acho adequado que” e a simplificação da escala Likert para três pontos: (1) discordo; (2) não concordo nem discordo; (3) concordo (Material Suplementar - Quadro S5; https://cadernos.ensp.fiocruz.br/static//arquivo/suppl-e00053925_4814.pdf).

Também foi adicionada uma pergunta aberta sobre a qualidade e variedade dos alimentos na cantina, baseada em questões encontradas nos questionários de Hermans et al. ^24^ (“*What score would you give your school canteen?*” [Que nota você daria para a cantina da sua escola?] e “*How important is the school canteen to you?*” [Quão importante é a cantina da escola para você?]) e de Gosliner et al. ^23^ (“*How do you rate the Healthy School Canteen Program initiative*?” [Como você avalia a iniciativa do Programa de Cantina Escolar Saudável?]). Após essas mudanças, ocorreu uma segunda rodada de avaliação com os adolescentes, que não apontou a necessidade de alterações adicionais.

### Questões éticas

Este estudo seguiu os princípios da *Declaração de Helsinki* e as diretrizes das *Resoluções nº 466/2012* e *nº 510/2016* do Conselho Nacional de Saúde. Foi aprovado pelo Comitê de Ética em Pesquisa da Ensp/FIOCRUZ (CAAE nº 60444322.8.0000.5240). Todos os participantes assinaram termos de consentimento ou assentimento livre e esclarecido.

### Análise descritiva dos resultados metodológicos

Foram calculadas taxas de elegibilidade, participação e recusa das escolas, bem como o percentual da amostra alcançada em cada porte. O número de escolas elegíveis foi utilizado como denominador das taxas de participação e recusa, pois todas foram convidadas para a pesquisa. Para os alunos, a taxa de participação foi estimada com base em uma média esperada de 30 alunos por turma e no total de turmas convidadas em cada escola (3), resultando em 90 alunos por escola.

Para descrever o perfil da participação obtida, foram calculadas as frequências absolutas e relativas dos aspectos administrativos das cantinas escolares e dos dados sociodemográficos dos alunos. Todos os cálculos foram realizados no RStudio, versão 4.3.2 (https://rstudio.com/).

## Resultados

A coleta de dados do primeiro ano do estudo *Impacto da Regulamentação do Ambiente Alimentar Escolar* estava prevista para ser concluída até dezembro de 2023, mas precisou ser estendida. O trabalho de campo foi finalizado em abril de 2024, em Niterói e Porto Alegre, e em maio, em Recife. Com isso, a segunda coleta, inicialmente planejada para o segundo semestre de 2024, foi adiada para o primeiro semestre de 2025.

Até dezembro de 2023, o Módulo Cantina havia alcançado somente 56,7% (n = 38) das escolas previstas em Niterói e 50% (n = 27) em Porto Alegre, apesar de todas as escolas já terem sido triadas quanto à elegibilidade e todas as escolas elegíveis terem sido convidadas a participar. Em Recife, onde o número de escolas era maior, 79,2% (n = 262) haviam sido triadas, e a amostra obtida correspondia a 52,9% do planejado (n = 73).

Diante desse cenário, a coleta de dados foi prorrogada. Entre março e maio de 2024, as escolas de Niterói e Porto Alegre que não haviam respondido em 2023, foram novamente convidadas. Em Recife, as escolas ainda não triadas foram contatadas, e as elegíveis, convidadas a participar.

Ao final, 201 escolas aderiram ao Módulo Cantina: 48 em Niterói, 32 em Porto Alegre e 121 em Recife. A taxa de participação foi menor em Porto Alegre (47,8%), seguida por Niterói (53,9%) e Recife (69,9%), resultando em 71,6%, 59,3% e 87,7% dos tamanhos amostrais previstos, respectivamente (Tabela 1). Em Porto Alegre, a adesão foi maior entre escolas pequenas (51-200 alunos) (66,7%) e grandes (> 1.000 alunos) (64,7%). Em Recife, escolas pequenas apresentaram maior participação (82%), enquanto apenas 41,7% das grandes aderiram (Tabela 1).

**Tabela 1:** Status final da coleta de dados do Módulo Cantina do estudo *Impacto da Regulamentação do Ambiente Alimentar Escolar*. Niterói (Rio de Janeiro), Porto Alegre (Rio Grande do Sul) e Recife (Pernambuco), Brasil, 2023/2024.

Cidade/Porte escolar (alunos)	Universo amostral *	Tamanho amostral **	Taxa de elegibilidade ***	Taxa de participação ^#^	Taxa de recusa ^##^	Amostra obtida ^###^
	n	n	n (%)	n (%)	n (%)	n (%)
Total	562	259	329 (58,5)	201 (61,1)	128 (38,9)	201 (77,6)
Niterói						
51-200	80	40	42 (52,5)	22 (52,4)	20 (47,6)	22 (55,0)
201-500	34	20	32 (94,1)	16 (50,0)	16 (50,0)	16 (80,0)
501-1.000	11	5	11 (100,0)	6 (54,5)	5 (45,4)	6 (120,0)
> 1.000	4	2	4 (100,0)	4 (100,0)	0 (0,0)	4 (200,0)
Subtotal	129	67	89 (69,0)	48 (53,9)	41 (46,0)	48 (71,6)
Porto Alegre						
51-200	31	5	6 (19,3)	4 (66,7)	2 (33,3)	4 (80,0)
201-500	27	14	18 (66,7)	7 (38,9)	11 (61,1)	7 (50,0)
501-1.000	27	21	26 (96,3)	10 (45,4)	16 (61,5)	10 (47,6)
> 1.000	17	14	17 (100,0)	11 (64,7)	6 (35,3)	11 (78,6)
Subtotal	102	54	67 (65,0)	32 (47,8)	35 (52,2)	32 (59,3)
Recife						
51-200	197	54	61 (31,0)	50 (82,0)	11 (22,0)	50 (92,6)
201-500	90	57	70 (77,8)	49 (70,0)	21 (30,0)	49 (87,7)
501-1.000	32	19	30 (93,8)	17 (56,7)	13 (43,3)	17 (89,5)
> 1.000	12	8	12 (100,0)	5 (41,7)	7 (58,3)	5 (62,5)
Subtotal	331	138	173 (52,3)	121 (69,9)	52 (30,1)	121 (87,7)

Nota: todas as escolas do Catálogo de Escolas do Instituto Nacional de Estudos e Pesquisas Educacionais Anísio Teixeira (INEP) foram triadas quanto à elegibilidade.

* Universo amostral obtido no Catálogo de Escolas do INEP; excluídas instituições que não são escolas (p.ex.: beneficentes) ou inoperantes;

** Tamanho amostral definido a partir do Catálogo de Escolas do INEP;

*** Percentual de escolas com cantina, tendo o universo amostral como denominador;

^#^ Escolas em que a pesquisa foi realizada, tendo a quantidade de escolas elegíveis como denominador;

^##^ Escolas em que a participação na pesquisa foi recusada ou que não responderam ao convite, tendo a quantidade de escolas elegíveis como denominador;

^###^ Percentual calculado, tendo o tamanho amostral como denominador.

No Módulo Ambulante, até dezembro de 2023, haviam sido identificados apenas um vendedor em Recife, dois em Porto Alegre e nenhum em Niterói. Dado o número insuficiente de observações, os dados desse módulo não foram apresentados.

**Figura 1:**
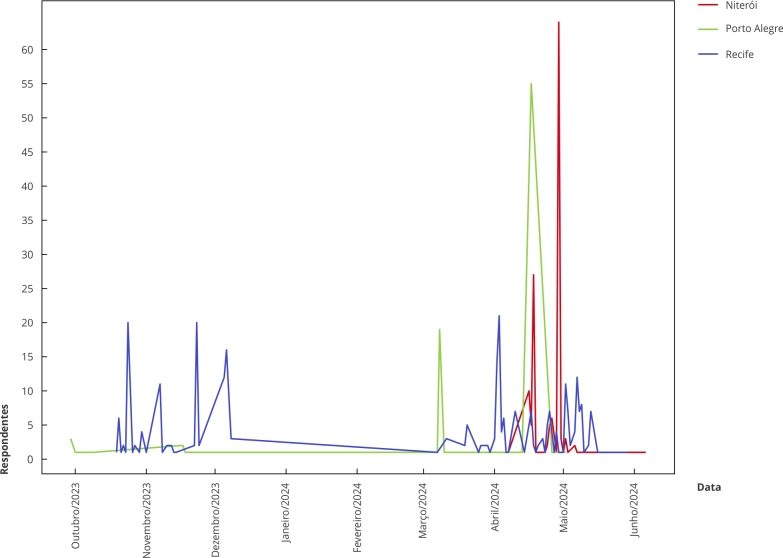
Participação de estudantes no Módulo Aluno do estudo *Impacto da Regulamentação do Ambiente Alimentar Escolar* ao longo da coleta de dados. Niterói (Rio de Janeiro), Porto Alegre (Rio Grande do Sul) e Recife (Pernambuco), Brasil, 2023/2024.

A adesão das escolas ao Módulo Aluno representou um desafio adicional. Para aumentar o engajamento, ainda em 2023, o *kit* digital foi substituído por um fôlder impresso entregue aos estudantes, com informações sobre a pesquisa e códigos QR para consentimento dos responsáveis e assentimento dos alunos. A mudança gerou discreto aumento de respostas em Recife, mas a participação em Niterói e Porto Alegre foi quase nula nesse período (Figura 1). Na tentativa de obter uma amostra suficientemente grande, ainda que não probabilística, optou-se por substituir o delineamento em três estágios por uma amostragem por conveniência. Assim, em 2024, todas as escolas que participaram do Módulo Cantina foram convidadas a compor a amostra do Módulo Aluno.

Apesar da adoção da amostragem por conveniência, o tamanho amostral calculado não foi atingido. Ao final, foram obtidas respostas de 532 estudantes de 46 escolas. Recife concentrou o maior número de escolas (n = 36) e respondentes (n = 301), embora com a menor taxa de participação (9,2%), comparada a 19,6% em Niterói e 20% em Porto Alegre. A maioria dos estudantes de Recife frequentava escolas de pequeno porte (n = 151) ou com 201-500 alunos (n = 98) (Tabela 2). A taxa de participação total foi de 12,9%, variando de 9,2% em Recife a 20% em Porto Alegre.

**Tabela 2:** Participação de escolas e de estudantes no Módulo Aluno do estudo *Impacto da Regulamentação do Ambiente Alimentar Escolar*. Niterói (Rio de Janeiro), Porto Alegre (Rio Grande do Sul) e Recife (Pernambuco), Brasil, 2023/2024.

Cidade/Porte escolar (alunos)	Subamostra de escolas	Escolas participantes *	Número estimado de alunos convidados **	Taxa de participação ***
	n	n	n	n (%)
Total	45	46	4.140	532 (12,8)
Niterói				
51-200	8	1	90	7 (7,8)
201-500	4	4	360	95 (26,4)
501-1.000	2	3	270	39 (14,4)
> 1.000	1	0	0	0 (0,0)
Subtotal	15	8	720	141 (19,6)
Porto Alegre				
51-200	4	2	180	5 (2,8)
201-500	4	1	90	26 (28,9)
501-1.000	4	0	0	0 (0,0)
> 1.000	3	2	180	59 (32,8)
Subtotal	15	5	450	90 (20,0)
Recife				
51-200	8	16	1.440	151 (10,5)
201-500	4	12	1.080	98 (9,1)
501-1.000	2	8	720	52 (7,2)
> 1.000	1	0	0	0 (0,0)
Subtotal	15	36	3.240	301 (9,2)

* Número excede o tamanho amostral planejado porque foi adotada amostragem por conveniência durante o estudo;

** Estimativa calculada com base nos quantitativos considerados no cálculo amostral original (três turmas com, aproximadamente, 30 alunos em cada escola participante);

*** Taxa calculada, tendo o número estimado de alunos convidados como denominador.

### Caracterização das cantinas e alunos

A maioria das cantinas é terceirizada, com menos de cinco funcionários e atende até 100 clientes por dia. O lanche é a principal refeição oferecida. Porto Alegre se destaca com 90,6% de cantinas terceirizadas, maior oferta de café da manhã (75%) e almoço (78,1%), mais cantinas com 5 a 10 funcionários (25%) e atendimento a mais de 200 clientes por dia (46,9%) (Tabela 3).

**Tabela 3:** Aspectos administrativos das cantinas de escolas da rede privada, com Ensino Fundamental ou Médio, participantes do Módulo Cantina do estudo *Impacto da Regulamentação do Ambiente Alimentar Escolar*. Niterói (Rio de Janeiro), Porto Alegre (Rio Grande do Sul) e Recife (Pernambuco), Brasil, 2023/2024.

Variáveis	Niterói (n = 49)		Porto Alegre (n = 32)		Recife (n = 124)	
	n	%	n	%	n	%
Tipo de administração						
Terceirizada	29	59,2	29	90,6	65	52,8
Própria	19	38,8	2	6,2	58	47,2
Outra	1	2,0	1	3,1	0	-
Número de funcionários						
< 5	48	98,0	22	68,8	105	85,4
5-10	1	2,0	8	25,0	15	12,2
11 ou mais	0	-	3	6,2	3	2,4
Número médio de clientes atendidos						
≤ 100	27	55,1	12	37,5	84	68,3
101-200	11	22,4	5	15,6	19	15,4
> 200	11	22,4	15	46,9	20	16,3
Refeições ofertadas						
Café da manhã	6	6,1	24	75,0	16	13,0
Almoço	11	22,4	25	78,1	39	31,7
Lanche	48	98,0	32	99,2	122	99,2
Jantar	2	4,1	2	6,2	2	1,6

A maior parte da amostra de alunos foi do sexo feminino (57,3%). A faixa etária predominante em todas as cidades foi de 12 a 13 anos, enquanto os alunos de 16 a 17 anos representaram a menor proporção. A maioria da amostra foi composta por brancos, com a maior proporção em Porto Alegre (81,1%) em comparação a Recife (40,5%). Porto Alegre apresentou uma proporção de pardos (10%) inferior a de Recife (44,2%). A maioria dos participantes tinha mães com Ensino Superior completo, sendo essa proporção maior em Porto Alegre (77,8%) do que em Niterói (50,4%) e Recife (45,9%) (Tabela 4).

**Tabela 4:** Aspectos sociodemográficos de estudantes participantes do Módulo Aluno do estudo *Impacto da Regulamentação do Ambiente Alimentar Escolar*. Niterói (Rio de Janeiro), Porto Alegre (Rio Grande do Sul) e Recife (Pernambuco), Brasil, 2023/2024.

Variáveis	Total (n = 532)		Niterói (n = 141)		Porto Alegre (n = 90)		Recife (n = 301)	
	n	%	n	%	n	%	n	%
Sexo								
Masculino	227	42,7	64	45,4	46	51,1	117	38,8
Feminino	305	57,3	77	54,6	44	48,9	184	61,1
Faixa etária (anos)								
12-13	283	53,2	75	53,2	47	52,2	161	53,5
14-15	203	38,2	57	40,4	41	45,6	105	34,9
16-17	46	8,6	9	6,4	2	2,2	35	11,6
Cor ou raça								
Branca	272	51,1	77	54,6	73	81,1	122	40,5
Parda	181	34,0	39	27,7	9	10,0	133	44,2
Preta	62	11,6	21	14,9	6	6,7	35	11,6
Amarela	12	2,3	3	2,1	1	1,1	8	2,7
Indígena	5	0,9	1	0,7	1	1,1	3	1,0
Escolaridade materna								
Nenhuma instrução ou Ensino Fundamental incompleto	13	2,4	3	2,1	0	-	10	3,3
Ensino Fundamental completo ou Ensino Médio incompleto	21	4,0	7	5,0	3	3,3	11	3,7
Ensino Médio completo ou Ensino Superior incompleto	152	28,6	40	28,4	6	6,7	106	35,2
Ensino Superior completo	279	52,4	71	50,4	70	77,8	138	45,9
Não sabe	67	12,6	20	14,2	11	12,2	36	12,0

## Discussão

O primeiro ano do estudo *Impacto da Regulamentação do Ambiente Alimentar Escolar* enfrentou desafios que impactaram a taxa de participação. O Módulo Cantina foi concluído com sucesso, apesar do tamanho amostral inferior ao pretendido. No Módulo Ambulante, não foram encontrados pontos de venda suficientes para compor uma amostra viável para análise. O Módulo Aluno não alcançou a amostra necessária mesmo após flexibilização do delineamento amostral.

### Módulo Cantina

Esperava-se que o trabalho de campo fosse concluído em menos tempo, uma vez que, no Caeb, o tamanho amostral planejado nessas cidades foi atingido em poucos meses ^25^. No entanto, houve forte resistência à participação. Recusas ocorreram devido a percepções negativas sobre a origem da pesquisa, vista por alguns cantineiros como associada a uma agenda política progressista. Além disso, equipes diretivas de algumas escolas recusaram por já terem colaborado com o Caeb no ano anterior. Em Niterói e Porto Alegre, a resistência pode estar relacionada ao receio de que a visita fosse associada à fiscalização das cantinas, especialmente após a repercussão da *Lei nº 3.766/2023* em Niterói ^26^ e da *Lei nº 7.987/2023* no Rio de Janeiro ^27^, que proíbem a venda e publicidade de alimentos ultraprocessados em escolas. Em Niterói, esse receio pode ter sido intensificado pelo uso de dados do Caeb por uma OSCIP em uma campanha para angariar apoio à aprovação da Lei. A campanha incluiu ações de *advocacy* junto aos poderes Executivo e Legislativo, ações on-line, divulgação em espaços públicos, bancas de jornal, ônibus, barcas e elevadores de prédios comerciais, além de diálogos com gestores escolares sobre a nova legislação. Diante desse cenário, a segunda coleta exigirá estratégias adicionais para manter ou ampliar a participação.

A divulgação do site e das reportagens sobre o estudo teve impacto limitado na receptividade à pesquisa, mas, em Niterói, houve êxito quando as visitas foram agendadas com o objetivo primário de orientar sobre a recém-aprovada Lei e entregar o recém-publicado *Guia Prático para uma Cantina Saudável* ^28^, elaborado com base na experiência do Caeb, para auxiliar na adaptação às novas normas. Então, somente durante as visitas, as escolas eram convidadas a participar da pesquisa. Em Recife, apesar do maior número de escolas a serem avaliadas e do esforço adicional necessário para cobrir esse volume dentro do mesmo prazo das outras cidades, a taxa de participação foi superior. Esse resultado pode ser atribuído, em parte, à ausência de legislação local vigente ^9^, o que fez com que os gestores escolares não vissem necessidade de temer fiscalização, além de um ambiente culturalmente mais receptivo.

Para melhorar a participação na segunda coleta, será fundamental divulgar antecipadamente o propósito da repetição da pesquisa. Nesse sentido, será buscado apoio para divulgação na mídia local, além de parcerias com sindicatos representantes das instituições de ensino locais.

Embora todas as escolas tenham sido convidadas, o tamanho amostral estimado não foi alcançado, o que pode comprometer o poder estatístico para detectar as relações esperadas. Em análises futuras, a ausência de diferenças estatisticamente significativas deve ser interpretada com cautela, pois pode refletir limitações amostrais e não a inexistência de diferenças reais. Também deve-se considerar que a alta taxa de recusas pode ter introduzido viés de seleção, distorcendo os resultados. Por exemplo, se as cantinas com maior oferta de alimentos não saudáveis tendem a recusar a participação, a prevalência de alimentos ultraprocessados pode ser subestimada. Além disso, é possível que escolas menores, com estrutura limitada para o preparo e armazenamento de alimentos perecíveis, apresentem maior disponibilidade desses alimentos. A alta participação de escolas de pequeno porte em Recife pode, portanto, gerar uma percepção de cenário mais negativo do que o real.

Como todas as possibilidades de reposição foram esgotadas e não foi possível obter uma amostra proporcional aos portes escolares, conforme o delineamento inicial, a pós-estratificação será empregada para corrigir eventuais sobre ou sub-representações desses estratos na amostra, visando aprimorar a validade externa e a generalização dos resultados ^29^. Com esse procedimento, a validade interna também poderá ser parcialmente aprimorada, mas o viés de auto-seleção não será eliminado, pois o porte escolar pode não capturar todas as fontes de viés.

### Módulo Ambulante

Foram encontrados poucos vendedores ambulantes em comparação ao Caeb, que, em 2022, entrevistou 69 ambulantes em Recife e 11 em Porto Alegre ^25^. Embora o Caeb tenha contratado uma empresa para a coleta de dados e nossa equipe tenha sido selecionada e treinada diretamente, a logística de coleta foi semelhante, e não houve alterações nas legislações locais que pudessem impactar a rotina dos ambulantes. Ressalta-se, contudo, que a amostra é de conveniência e foi coletada em uma única oportunidade. A presença desses trabalhadores pode variar conforme o horário da entrevista e as condições climáticas, tornando os cenários possivelmente distintos. Assim, tentaremos novamente obter uma amostra viável na segunda etapa do estudo.

### Módulo Aluno

Foi ainda mais desafiador obter a anuência das escolas para o Módulo Aluno. Visando aumentar a credibilidade da pesquisa, pesquisadores da coordenação local assumiram os convites, em alguns casos, indo pessoalmente às instituições. Mesmo assim, apenas um número reduzido de escolas em Porto Alegre e Niterói aceitaram participar, o que sugere que abordagens sem vínculos prévios podem não ser bem-sucedidas em pesquisas com dados sensíveis de adolescentes.

Embora se previsse uma taxa de participação inferior a de pesquisas escolares presenciais, como o *Estudo dos Riscos Cardiovasculares em Adolescentes* (ERICA) (76,2%) ^30^, o resultado ficou abaixo da média de 44,1% observada em pesquisas on-line ^31^. Isso pode ter ocorrido porque, além da necessidade de aceitação por parte da escola, a natureza on-line e assíncrona da pesquisa exigia que os alunos se engajassem autonomamente para obter o consentimento dos responsáveis e preencher o questionário em casa.

Diante da dificuldade de recrutamento, adotou-se a amostragem por conveniência para tentar atingir o número mínimo de participantes. Ainda assim, a amostra final foi insuficiente para estimar com confiança os parâmetros desejados, tornando as análises essencialmente exploratórias. Contudo, o desenvolvimento do *Questionário de Percepção sobre a Cantina Escolar* representa uma contribuição importante desse trabalho para o campo de estudo sobre o ambiente alimentar. Apesar do curto prazo para a construção do instrumento, o processo incluiu revisão da literatura, validação por especialistas e pré-testes. Cabe destacar, no entanto, que a avaliação contou apenas com especialistas da própria equipe de pesquisa e que o instrumento ainda não foi submetido à avaliação de confiabilidade ou validade. Assim, essas limitações devem ser sanadas em versões futuras do instrumento, de modo a garantir sua robustez metodológica. Ainda assim, os dados coletados podem ser tomados como um estudo-piloto para avaliar a estrutura interna dos itens e aprimorar o instrumento para pesquisas futuras.

As percepções dos alunos sobre a cantina escolar e as regulamentações vigentes podem fornecer *insights* valiosos para aprimorar a formulação e implementação de políticas públicas. Por isso, para garantir o sucesso do Módulo Aluno, a segunda etapa do estudo adotará uma abordagem qualitativa. Serão realizados grupos focais com uma amostra propositalmente selecionada, utilizando roteiros baseados nos itens do questionário de percepção e adaptados ao contexto regulatório de cada localidade.

## Considerações finais

Este artigo apresentou os três módulos do estudo *Impacto da Regulamentação do Ambiente Alimentar Escolar*, as adversidades enfrentadas em sua primeira etapa e as estratégias adotadas para superá-las. A experiência acumulada reforça a importância de adaptar abordagens de pesquisa às especificidades culturais e políticas de cada contexto, especialmente em estudos na escola. Estratégias de engajamento, como divulgação antecipada, apoio de organizações locais e a oferta de benefícios concretos, como orientações para implementar mudanças nas cantinas, podem fortalecer a colaboração e o envolvimento das instituições.

Os dados deste estudo, juntamente aos dados previamente coletados pelo Caeb, fornecem uma base para futuras análises das relações entre mudanças no ambiente escolar e o contexto regulatório ao longo de três anos que fornecerão subsídios para o aprimoramento de políticas públicas em diferentes cidades e esferas governamentais. Além disso, a seleção das cidades de Niterói, Porto Alegre e Recife, orientada por demandas da sociedade civil, ressalta a importância da colaboração entre academia e sociedade.

## Data Availability

Os dados de pesquisa estão disponíveis mediante solicitação à autora de correspondência.

## References

[1] Food and Agriculture Organization of the United Nations (2019). School food and nutrition framework.

[2] Gabriel CG, Ricardo GD, Ostermann RM, Corso ACT, Assis MAA, Pietro PFD (2012). Regulamentação da comercialização de alimentos no ambiente
escolar análise dos dispositivos legais brasileiros que buscam a alimentação
saudável. Rev Inst Adolfo Lutz.

[3] Brasil (2009). Lei nº 11.947, de 16 de junho de 2009. Dispõe sobre o atendimento
da alimentação escolar e do Programa Dinheiro Direto na Escola aos alunos da
educação básica.. Diário Oficial da União.

[4] Instituto Brasileiro de Geografia e Estatística (2021). Pesquisa Nacional de Saúde do Escolar: 2019.

[5] Azeredo CM, De Rezende LFM, Canella DS, Claro RM, Peres MFT, Luiz ODC (2016). Food environments in schools and in the immediate vicinity are
associated with unhealthy food consumption among Brazilian
adolescents. Prev Med.

[6] Carmo AS, Assis MM, Cunha CF, Oliveira TRPR, Mendes LL (2018). The food environment of Brazilian public and private
schools. Cad Saúde Pública.

[7] Rocha LL, Cordeiro NG, Jardim MZ, Kurihayashi AY, Gentil PC, Russo GC (2023). Do Brazilian regulatory measures promote sustainable and healthy
eating in the school food environment. BMC Public Health.

[8] Canuto R, Clark SGF, Borges LD, Castro PCP, Tavares LF, Cardoso LO (2025). Aspectos metodológicos do estudo Comercialização de Alimentos em
Escolas Brasileiras.. Cad Saúde Pública.

[9] Câmara Municipal de Recife (2022). Substitutivo nº 1 ao Projeto de Lei Ordinária nº 172/2022. Proíbe a
venda e a distribuição de bebidas açucaradas e de alimentos ultraprocessados
nas escolas públicas e privadas do município do Recife e dá outras
providências.

[10] Niterói (2023). Lei Ordinária nº 3766 de 5 de janeiro de 2023. Altera a Lei de nº
2659, de 19 de novembro de 2009, proíbe a comercialização, a aquisição, a
confecção, a distribuição e a publicidade de produtos que contribuem para a
obesidade infantil e dá outras providências.. Diário Oficial do Município de Niterói.

[11] Instituto Brasileiro de Geografia e Estatística (2016). Pesquisa Nacional de Saúde do Escolar: 2015.

[12] Haldane JBS (1945). On a method of estimating frequencies. Biometrika.

[13] Wu MJ, Zhao K, Fils-Aime F (2022). Response rates of online surveys in published research a
meta-analysis. Comput Hum Behav Rep.

[14] Botelho LV, Cardoso LO Protocolo de campo do estudo Impacto da Regulamentação do Ambiente
Alimentar Escolar: avaliação em três cidades brasileiras..

[15] Universidade Federal do Rio de Janeiro Comercialização de Alimentos em Escolas Brasileiras..

[16] Martins ML, Botelho LV, Cardoso LO Manual de cadastro e uso do REDCap Mobile App: Caeb
Longitudinal..

[17] Martins ML, Botelho LV, Cardoso LO Vídeo instrutivo para cadastro e uso do REDCap Mobile App: Caeb
Longitudinal..

[18] Monteiro C, Cannon G, Levy R, Moubarac JC (2016). The food system NOVA. The star shines bright. World Nutr.

[19] Instituto Brasileiro de Geografia e Estatística (2021). Pesquisa Nacional de Saúde.

[20] Departamento de Análise em Saúde e Vigilância de Doenças Não
Transmissíveis. Secretaria de Vigilância em Saúde. Ministério da
Saúde (2022). Vigitel Brasil 2021: estimativas sobre frequência e distribuição
sociodemográfica de fatores de risco e proteção para doenças crônicas nas
capitais dos 26 estados brasileiros e no Distrito Federal em 2021.

[21] Botelho LV, Borges LD, Martins ML Relatório de pesquisa para o estudo Comercialização de Alimentos em
Escolas Brasileiras (Caeb): revisão de escopo para identificar instrumentos
de avaliação do ambiente alimentar escolar percebido por
estudantes..

[22] Eagly AH, Chaiken S (2011). The psychology of attitudes.

[23] Gosliner W, Madsen KA, Woodward-Lopez G, Crawford PB (2011). Would students prefer to eat healthier foods at
school. J Sch Health.

[24] Hermans RCJ, De Bruin H, Larsen JK, Mensink F, Hoek AC (2017). Adolescents' responses to a school-based prevention program
promoting healthy eating at school. Front Public Health.

[25] Mendes LL, Canuto R, Tavares LF, Cardoso LO, Castro PCP, Borges LD (2024). Relatório do Estudo Comercialização de Alimentos em Escolas Brasileiras
(Caeb): dados das cantinas escolares..

[26] Torres E (2023). Lei que proíbe venda de alimentos ultraprocessados em escolas
entra em vigor em Niterói.. O Globo.

[27] Perez B (2023). Escolas particulares serão principais afetadas por legislação que
restringe ultraprocessados no Rio.. O Dia.

[28] Instituto Desiderata (2023). Guia prático para uma cantina saudável..

[29] Silva PLN (2004). Calibration estimation! When and why, how much and how.

[30] Silva TLN, Klein CH, Souza AM, Barufaldi LA, Abreu GA, Kuschnir MCC (2016). Response rate in the Study of Cardiovascular Risks in Adolescents
- ERICA. Rev Saúde Pública.

[31] De Boni RB (2020). Websurveys nos tempos de COVID-19. Cad Saúde Pública.

