# Supplementary material for: Impact of Regulation of the School Food Environment: methodological aspects and participation in the first year of follow-up
Source: Cad Saude Publica. 2025 Nov 7;41(10):e00053925. [Article in Portuguese] doi: 10.1590/0102-311XPT053925 (PMC12600018; doi:10.1590/0102-311XPT053925)
Supplement: Supplementary Material [file 1678-4464-csp-41-10-PT053925-s.pdf]

MATERIAL SUPLEMENTAR

Quadro S1 Descrição dos dispositivos legais para promoção da alimentação adequada e saudável na escola vigentes ou em discussão para Recife (PE), Niterói (RJ) e Porto Alegre (RS), 2024 (continua).

Localidade de incidência	Esfera	Ano de publicação	Dispositivo	Número de identificação	Descrição do dispositivo
Recife	Municipal	02/05/2022	Projeto de Lei Ordinária	172	Proíbe a venda e a distribuição de bebidas açucaradas e de alimentos ultraprocessados nas escolas públicas e privadas do município do Recife e dá outras providências.
	Municipal	29/05/2022	Substitutivo ao Projeto de Lei Ordinária n.º 172/2022	1	Proíbe a venda e a distribuição de bebidas açucaradas e de alimentos ultraprocessados nas escolas públicas e privadas do município do Recife e dá outras providências.
Niterói	Estadual	11/01/2005	Lei	4508	Proíbe a comercialização, aquisição, confecção e distribuição de produtos que colaborem para a obesidade infantil, em bares, cantinas e similares instalados em escolas públicas e privadas do estado do Rio de Janeiro.
	Municipal	19/11/2009	Lei	2659	Proíbe a comercialização, a aquisição, a confecção, a distribuição e a publicidade de produtos que contribuem para a obesidade infantil e dá outras providências.
	Municipal	05/01/2023	Lei	3766	Altera a Lei de n.º 2659, de 19 de novembro de 2009, proíbe a comercialização, a aquisição, a confecção, a distribuição e a publicidade de produtos que contribuem para a obesidade infantil e dá outras providências.
	Municipal	21/07/2023	Lei	3800	Revoga os artigos 4º e 5º da lei municipal n.º 3.766/2023.
	Municipal	06/06/2024	Decreto	15457	Regulamenta a Lei n.º 2659/2009 que proíbe a comercialização, a aquisição, a confecção, a distribuição e a publicidade de produtos que contribuem para a obesidade infantil e dá outras providências.

Quadro S1 Descrição dos dispositivos legais para promoção da alimentação adequada e saudável na escola vigentes ou em discussão para Recife (PE), Niterói (RJ) e Porto Alegre (RS), 2024 (conclusão).

Localidade de incidência	Esfera	Ano de publicação	Dispositivo	Número de identificação	Descrição do dispositivo
Porto Alegre	Municipal	24/01/2007	Lei	10167	Estabelece, no município de Porto Alegre, normas para o controle da comercialização de produtos alimentícios e de bebidas nos bares e nas cantinas das escolas públicas e privadas e dá outras providências.
	Estadual	16/08/2008	Lei	13027	Dispõe sobre a comercialização de lanches e de bebidas em escolas no âmbito do Estado do Rio Grande do Sul e dá outras providências.
	Estadual	30/07/2018	Lei	15216	Dispõe sobre a promoção da alimentação saudável e proíbe a comercialização de produtos que colaborem para a obesidade, diabetes, hipertensão, em cantinas e similares instalados em escolas públicas e privadas do Estado do Rio Grande do Sul.
	Estadual	17/01/2020	Decreto	54994	Regulamenta a Lei n.º 15.216, de 30 de julho de 2018, que dispõe sobre a promoção da alimentação saudável e proíbe a comercialização de produtos que colaborem para a obesidade, diabetes e hipertensão em cantinas e similares instalados em escolas públicas e privadas do Estado do Rio Grande do Sul.

Fonte: Sistema de Apoio ao Processo Legislativo (SAPL)

Nota: Todos os dispositivos legais abrangem tanto escolas públicas quanto privadas.

Figura S1 Organograma funcional e atribuições no estudo Impacto da Regulamentação do Ambiente Alimentar Escolar.

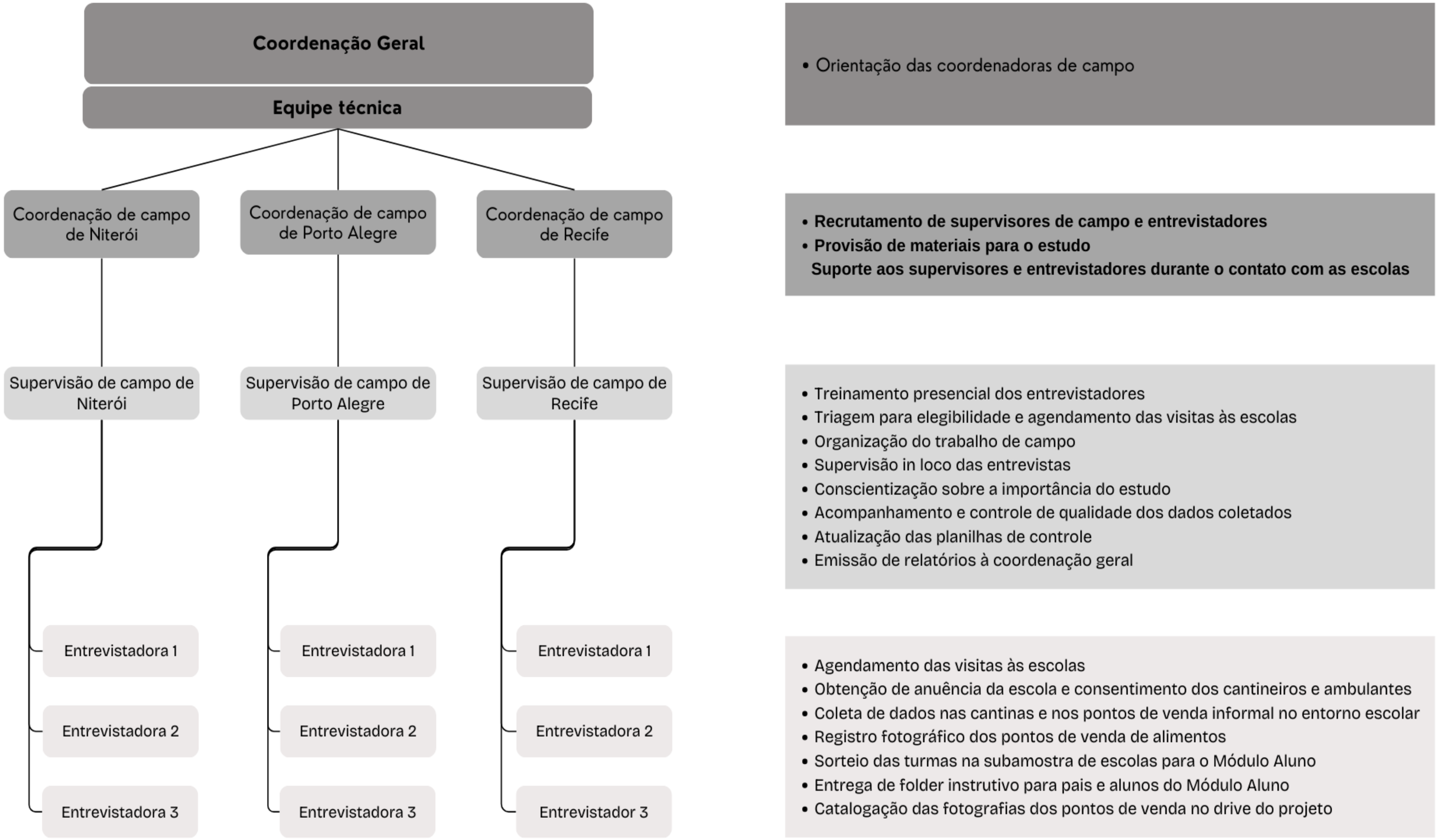

Quadro S2 Variáveis coletadas no Módulo Cantina do estudo Impacto da Regulamentação do Ambiente Alimentar Escolar.

Blocos	Dados Coletados
Identificação e caracterização	Tipo de administração<br>Número de funcionários<br>Número médio de clientes atendidos<br>Presença de nutricionista<br>Local de produção dos alimentos<br>Critério de seleção dos alimentos<br>Comercialização de alimentos para fins especiais<br>Venda de alimentos por outros membros da comunidade escolar<br>Venda de refeição<br>Disponibilidade de produtos de adição<br>Produtos de adição disponíveis<br>Disponibilidade de informações alimentares e nutricionais<br>Disponibilidade de cardápio<br>Refeições para as quais cardápio está disponível<br>Formas de disponibilização do cardápio<br>Menor preço prato feito<br>Menor preço kg<br>Formas de pagamento aceitas<br>Recebimento de material de incentivo / patrocínio / apoio de fornecedores<br>Materiais recebidos<br>Empresas fornecedoras dos materiais<br>Oferta de combo<br>Oferta de promoções<br>Realização de ações de incentivo à alimentação saudável<br>Ações de incentivo à alimentação saudável realizadas<br>Presença de materiais educativos sobre alimentação saudável na cantina<br>Responsabilidade pelo desenvolvimento dos materiais educativos<br>Refeições oferecidas<br>Preço disponível para consulta<br>Formas de disponibilização do preço<br>Presença de estrutura para consumo no local<br>Proibição da venda de alimentos por parte da escola<br>Alimentos proibidos pela escola
Comercialização de alimentos	Alimentos e bebidas comercializados (listados em um checklist)<br>Variedade (n.º de itens diferentes, considerando os diversos sabores e marcas, para cada alimento ou bebida do checklist)<br>Tamanho do produto mais barato (g/mL/unidade), considerando cada alimento ou bebida do checklist<br>Menor preço de cada alimento ou bebida do checklist<br>Oferta de combo contendo cada alimento ou bebida do checklist<br>Oferta de promoção contendo cada alimento ou bebida do checklist
Publicidade dos alimentos comercializados	Presença de publicidade das seguintes formas, para cada alimento ou bebida comercializado:<br>1. Banner/cartaz do fornecedor

	2. Banner/cartaz do estabelecimento<br>3. Vestimenta<br>4. Réplica do produto<br>5. Cardápio<br>6. Embalagem<br>7. Painel/televisão<br>8. Folder<br>9. Displays<br>10. Brindes<br>11. Aplicativo da escola/cantina

Quadro S3 Subgrupos alimentares componentes da lista de verificação do Instrumento para Avaliação da Comercialização de Alimentos em Cantinas de Escolas.

Alimentos *in natura* ou minimamente processados, processados e preparações culinárias à base desses alimentos	Alimentos ultraprocessados e preparações culinárias à base desses alimentos
Água mineral (com ou sem gás)	Refresco
Água de coco	Néctar de fruta em caixinha, lata ou garrafa
Suco natural de fruta	Chá pronto para beber
Suco 100% natural de caixinha, lata ou garrafa	Bebida láctea e iogurte com sabor
Café	Bebida à base de soja
Chá natural	Refrigerante comum
Leite batido com fruta/ vitamina	Refrigerante zero, light, diet
Fruta fresca	Energético
Fruta seca/ desidratada	Isotônico
Salada de fruta simples	Salada de fruta com toppings
Açaí sem açúcar ou xarope	Açaí com açúcar ou xarope
Bolo de preparação culinária	Açaí com toppings
Doce a base de frutas ou legumes	Barra de cereais
Biscoito preparação culinária	Cereal matinal
Pão de queijo (preparação culinária própria)	Bolo ultraprocessado
Tapioca sem recheio ultraprocessado	Doce com ingredientes ultraprocessados
Pizza sem recheio ultraprocessado	Bombom ou chocolate
Salgado assado sem recheio ultraprocessado	Guloseima
Salgado frito sem recheio ultraprocessado	Picolé ou sorvete
Sanduíche sem recheio ultraprocessado	Biscoito doce com ou sem recheio ultraprocessado
Pipoca feita com grão natural doce ou salgada	Salgadinho de pacote, chips, biscoito/bolacha salgado
	Pão de queijo congelado ou mistura pronta
	Tapioca com recheio ultraprocessado
	Pizza com recheio ultraprocessado
	Salgado assado com recheio ultraprocessado
	Salgado frito com recheio ultraprocessado
	Sanduíche com recheio ultraprocessado
	Pipoca ultraprocessada
	Pipoca de pacote doce e/ou salgada

Quadro S4 Descrição do Questionário do Aluno aplicado no estudo Impacto da Regulamentação do Ambiente Alimentar Escolar.

Parte do questionário	Tipos de informações coletadas	Variáveis	Fonte das questões
Bloco 1:<br>Informações gerais	Características demográficas e socioeconômicas (17 itens)	Sexo, idade, presença de bens domésticos, características da habitação e aspectos socioeconômicos dos residentes para cálculo do Indicador Econômico Nacional.	PeNSE, 2019
	Antropometria (2 itens)	Peso e altura autorreferidos.	PNS, 2019
Bloco 2: Percepção sobre a cantina escolar	Aspectos relacionados à comercialização de alimentos na cantina (28 itens)	Frequência de uso da cantina, nota geral para os alimentos comercializados e percepção sobre diferentes aspectos do ambiente alimentar da escola: disponibilidade, variedade, preço, publicidade e qualidade de alimentos vendidos; educação alimentar e nutricional; envolvimento de membros da comunidade escolar.	PeNSE, 2019 (somente pergunta sobre frequência de uso da cantina).<br>Demais itens foram desenvolvidos para o próprio estudo.
Bloco 3: Alimentação	Práticas alimentares habituais (4 itens) e Recordatório de 24 horas para marcadores de consumo alimentar saudável (13 itens) e não saudável (13 itens)	Hábitos alimentares; Instrumentos simplificados compostos por questões sobre o consumo no dia anterior (sim ou não) de uma lista de subgrupos de alimentos *in natura* ou minimamente processados e de alimentos ultraprocessados.	VIGITEL 2021; PeNSE, 2019
Bloco 4: Atividade Física	Atividade física de lazer e Deslocamento ativo (10 itens)	Atividade física de transporte e lazer (frequência e tempo de duração) e uso de telas.	PeNSE, 2019

Quadro S5 Desenvolvimento do Questionário de Percepção do Ambiente Alimentar Escolar utilizado no Módulo Aluno no estudo Impacto da Regulamentação do Ambiente Alimentar Escolar (continua).

Item	Critérios avaliados por especialistas												Versão pré-teste [b]	Versão final [c]
	"O item é relevante."				"O item está claramente escrito e sem ambiguidade."				"As opções de respostas estão claramente escritas e sem ambiguidade."					
	Pontuação atribuída por cada especialista [a]													
	I	II	III	IV	I	II	III	IV	I	II	III	IV		
1) Você costuma comprar alimentos ou bebidas na cantina dentro da escola? (Não considerar a compra de água). [d]	5	5	5	5	5	5	5	5	5	4	3	4	Não modificado	Não modificado
Para cada afirmação abaixo, assinale em que medida você concorda ou discorda. [e]														
2) A minha escola sempre disponibiliza água, gratuita e de fácil acesso para beber.	5	5	5	5	5	5	5	5	5	5	5	4	Não modificado	Não modificado
3) A qualidade da água para beber da minha escola é boa.	5	4	3	5	5	4	5	5	5	5	3	4	Retirado	Ausente
4) É importante que a cantina da minha escola venda água mineral.	5	5	4	5	5	4	5	5	5	4	5	4	Não modificado	**Acho adequada a** venda de água mineral na cantina da minha escola.
5) É importante que a cantina da minha escola venda refrigerante.	5	5	4	5	5	5	5	5	5	5	5	4	Não modificado	**Acho adequada a** venda de refrigerante na cantina da minha escola.

Quadro S5 Desenvolvimento do Questionário de Percepção do Ambiente Alimentar Escolar utilizado no Módulo Aluno no estudo Impacto da Regulamentação do Ambiente Alimentar Escolar (continuação).

Item	Critérios avaliados por especialistas												Versão pré-teste [b]	Versão final [c]
	"O item é relevante."				"O item está claramente escrito e sem ambiguidade."				"As opções de respostas estão claramente escritas e sem ambiguidade."					
	Pontuação atribuída por cada especialista [a]													
	I	II	III	IV	I	II	III	IV	I	II	III	IV		
6) É importante que a cantina da minha escola venda bebidas como suco de fruta em caixinha ou lata (ex. Del Valle®, Tial®), isotônico (ex. Gatorade®, Power Ade®), energético (ex. Red Bull®, Monster®),guaraná natural (ex. Guaravita®, Guaracamp®) e chá pronto para beber (ex. Matte Leão®, Ice tea®).	5	5	4	5	5	5	5	5	5	5	5	4	Não modificado	**Acho adequada** a venda de bebidas como suco de fruta em caixinha ou lata (ex. Del Valle®), isotônico (ex. Gatorade®), energético (ex. Red Bull®), guaraná natural (Guaravita®) e chá pronto para beber (Matte Leão ®) na cantina da minha escola.
7) É importante que a cantina da minha escola venda doces, como balas, confeitos, chocolates, chicletes, bombons, pirulitos e outros.	5	5	4	5	5	5	5	5	5	5	5	4	Não modificado	**Acho adequada a** venda de doces, como balas, confeitos, chocolates, chicletes, bombons, pirulitos e outros na cantina da minha escola.
8) É importante que a cantina da minha escola venda salgadinho de pacote (chips) ou biscoitos/bolachas ou bolinho de pacote.	5	5	4	5	5	5	5	5	5	5	5	4	Não modificado	**Acho adequada a** venda de salgadinho de pacote (chips) ou biscoitos/bolachas ou bolinho de pacote na cantina da minha escola.
9) É importante que a cantina da minha escola venda bolo ou biscoito/bolacha caseiro (sem ser industrializado).	5	5	4	5	5	4	5	5	5	5	5	4	Não modificado	**Acho adequada a** venda de bolo ou biscoito/bolacha sem ser industrializado (feito na cantina/em casa) na cantina da minha escola.

Quadro S5 Desenvolvimento do Questionário de Percepção do Ambiente Alimentar Escolar utilizado no Módulo Aluno no estudo Impacto da Regulamentação do Ambiente Alimentar Escolar (continuação).

Item	Critérios avaliados por especialistas												Versão pré-teste [b]	Versão final [c]
	"O item é relevante."				"O item está claramente escrito e sem ambiguidade."				"As opções de respostas estão claramente escritas e sem ambiguidade."					
	Pontuação atribuída por cada especialista [a]													
	I	II	III	IV	I	II	III	IV	I	II	III	IV		
10) É importante que a cantina da minha escola venda suco natural da fruta.	5	5	4	5	5	5	5	5	5	5	5	4	Não modificado	**Acho adequada a** venda de suco natural da fruta na cantina da minha escola.
11) É importante que a cantina da minha escola venda frutas frescas ou salada de frutas frescas.	5	5	4	5	5	5	5	5	5	5	5	4	Não modificado	**Acho adequada a** venda de frutas frescas ou salada de frutas frescas na cantina da minha escola.
12) Na cantina da minha escola existe uma variedade grande de refrigerantes.	5	5	4	5	2	5	5	5	5	5	5	4	Na cantina da minha escola existem **várias opções de** refrigerantes.	**Acho adequado que existam** várias opções de refrigerantes na cantina da minha escola.
13) Na cantina da minha escola existe uma variedade grande de sabores de suco natural da fruta.	5	5	4	5	2	5	5	5	5	5	5	4	Na cantina da minha escola existem **várias opções de** sabores de suco natural da fruta.	**Acho adequado que existam** várias opções de sabores de suco natural da fruta na cantina da minha escola.
14) De uma maneira geral, acho caro os preços dos alimentos e bebidas vendidos na cantina da minha escola.	5	5	4	5	5	5	5	5	5	5	5	4	Não modificado	Não modificado
15) Acho caro comprar refrigerante na cantina da minha escola.	5	5	4	5	5	5	5	5	5	5	5	4	Não modificado	Não modificado
16) Acho caro comprar suco natural da fruta na cantina da minha escola.	5	5	4	5	5	5	5	5	5	5	5	4	Não modificado	Não modificado
17) Na cantina da minha escola é comum ter promoção de alimentos e bebidas.	5	5	4	5	2	5	5	5	5	5	5	4	Não modificado	Não modificado

Quadro S5 Desenvolvimento do Questionário de Percepção do Ambiente Alimentar Escolar utilizado no Módulo Aluno no estudo Impacto da Regulamentação do Ambiente Alimentar Escolar (continuação).

Item	Critérios avaliados por especialistas												Versão pré-teste [b]	Versão final [c]
	"O item é relevante."				"O item está claramente escrito e sem ambiguidade."				"As opções de respostas estão claramente escritas e sem ambiguidade."					
	Pontuação atribuída por cada especialista [a]													
	I	II	III	IV	I	II	III	IV	I	II	III	IV		
18) Na cantina da minha escola é comum ter a opção de combo (combinado de alimentos com bebidas).	5	5	4	5	2	5	5	5	5	5	5	4	Não modificado	Na cantina da minha escola é comum ter a opção de combo (**venda combinada** de alimentos junto com bebidas).
19) A minha escola desenvolve ações que incentivem a alimentação saudável.	5	4	5	5	4	4	5	4	5	5	5	4	A minha escola desenvolve **atividades** que incentivem a alimentação saudável	Não modificado
20) A cantina da minha escola influencia ou pode influenciar no que eu escolho para comer.	5	5	4	5	5	5	5	5	5	5	5	4	Não modificado	Não modificado
21) A minha escola pode facilitar (promover) a alimentação saudável para os alunos.	5	5	5	5	5	5	5	5	5	5	5	4	Não modificado	Não modificado
22) A cantina da minha escola é saudável.	5	5	4	5	5	5	5	4	5	5	5	4	**Os alimentos vendidos** na cantina da minha escola são saudáveis.	Não modificado
23) É importante a minha escola se preocupar com os alimentos que são vendidos na cantina.	5	4	4	5	5	4	5	5	5	5	5	4	Não modificado	Não modificado

Quadro S5 Desenvolvimento do Questionário de Percepção do Ambiente Alimentar Escolar utilizado no Módulo Aluno no estudo Impacto da Regulamentação do Ambiente Alimentar Escolar (continuação).

Item	Critérios avaliados por especialistas												Versão pré-teste [b]	Versão final [c]
	“O item é relevante.”				“O item está claramente escrito e sem ambiguidade.”				“As opções de respostas estão claramente escritas e sem ambiguidade.”					
	Pontuação atribuída por cada especialista [a]													
	I	II	III	IV	I	II	III	IV	I	II	III	IV		
24) É importante que meus pais e/ou responsáveis se preocupem com os alimentos que são vendidos na cantina.	5	4	4	5	5	4	5	5	5	5	5	4	Não modificado	Não modificado
25) Eu gostaria que a cantina da minha escola oferecesse opções de alimentos e bebidas mais saudáveis .	5	5	4	5	2	5	5	4	5	5	5	4	Eu gostaria que a cantina da minha escola **vendesse** opções de alimentos e bebidas mais saudáveis.	Não modificado
26) Na cantina da minha escola existem materiais educativos (murais, cartazes, banners, quadros etc.) sobre alimentação saudável.	5	5	4	5	5	4	5	4	5	5	5	4	Não modificado	Não modificado
27) Na cantina da minha escola existe publicidade de refrigerante. (OBS: a publicidade pode estar presente em banner/cartaz, vestimenta, réplica do produto/cardápio/embalagem/painel/televisão/folder/display e brindes)	5	5	4	5	4	4	5	5	5	5	5	4	Não modificado	Não modificado

Quadro S5 Desenvolvimento do Questionário de Percepção do Ambiente Alimentar Escolar utilizado no Módulo Aluno no estudo Impacto da Regulamentação do Ambiente Alimentar Escolar (conclusão).

Item	Critérios avaliados por especialistas												Versão pré-teste [b]	Versão final [c]
	"O item é relevante."				"O item está claramente escrito e sem ambiguidade."				"As opções de respostas estão claramente escritas e sem ambiguidade."					
	Pontuação atribuída por cada especialista [a]													
	I	II	III	IV	I	II	III	IV	I	II	III	IV		
28) Na cantina da minha escola existe publicidade de suco natural da fruta. (OBS: a publicidade pode estar presente em banner/cartaz, vestimenta, réplica do produto/cardápio/embalagem/painel/televisão/folder/display e brindes)	5	5	4	5	4	4	5	5	5	5	5	4	Não modificado	Não modificado

Nota: Os números I a IV representam os diferentes especialistas consultados.

[a] Pontuação correspondente a cada opção de resposta: (5) concordo totalmente; (4) concordo parcialmente; (3) não concordo nem discordo; (2) discordo parcialmente; (1) discordo totalmente.

[b] Versão após mudanças de acordo com a avaliação dos especialistas e que foi aplicada na primeira rodada de pré-teste com estudantes.

[b] Versão final utilizada no estudo, após incorporação das mudanças sugeridas pelos estudantes durante o pré-teste.

[c] Opções de resposta ao item 1: Sim, todos os dias; Sim, 3 a 4 dias por semana; Sim, 1 a 2 dias por semana; Raramente; Não costumo comprar alimentos ou bebidas na cantina.

[d] Opções de resposta aos itens 2 a 28: Concordo totalmente; concordo parcialmente; não concordo nem discordo; discordo parcialmente; discordo totalmente.
